# Bt Eggplant Project in Bangladesh: History, Present Status, and Future Direction

**DOI:** 10.3389/fbioe.2018.00106

**Published:** 2018-08-03

**Authors:** A. M. Shelton, M. J. Hossain, V. Paranjape, A. K. Azad, M. L. Rahman, A. S. M. M. R. Khan, M. Z. H. Prodhan, M. A. Rashid, R. Majumder, M. A. Hossain, S. S. Hussain, J. E. Huesing, L. McCandless

**Affiliations:** ^1^Department of Entomology, Cornell/NYSAES, Geneva, NY, United States; ^2^Feed the Future South Asia Eggplant Improvement Partnership, Dhaka, Bangladesh; ^3^Sathguru Management Consultants Pvt. Ltd., Hyderabad, India; ^4^Bangladesh Agricultural Research Institute, Gazipur, Bangladesh; ^5^USAID/BFS USDA/ARS OIRP, Research Division, Office of Agriculture Research & Policy, Washington, DC, United States; ^6^International Programs, Cornell University, Ithaca, NY, United States

**Keywords:** eggplant, brinjal, Bt crops, biotechnology, genetic engineering, fruit and shoot borer, *Leucinodes orbonalis*

## Abstract

The purpose of this article is to provide information on the history, accomplishments, and future direction of the Bt brinjal (eggplant) program in Bangladesh, formerly under the Agricultural Biotechnology Support Project II, now the South Asia Eggplant Improvement Partnership (SAEIP). The India-based Maharashtra Hybrid Seed Company (Mahyco) developed an eggplant expressing Cry1Ac (EE-1) for control of the eggplant fruit and shoot borer (EFSB). In a partnership among Mahyco, USAID, Sathguru Management Consultants and Cornell University EE-1 was provided to the Bangladesh Agricultural Research Institute (BARI) who bred it into local varieties. After regulatory approval, four varieties were distributed to 20 farmers who harvested Bt brinjal in 2014. Adoption in subsequent years has increased rapidly so that, in 2018, 27,012 farmers used this technology. This article provides background information on the process leading up to current adoption levels, the level of control of EFSB achieved and the economic benefits of Bt brinjal. Efforts on stewardship, farmer training and communication are discussed. In order to ensure the long-term future of the partnership, we discuss the need to enhance involvement of the private sector in the production and stewardship of Bt eggplant. Bt brinjal is the first genetically engineered crop to be commercially released in Bangladesh, and other GE crops are in the pipeline. Hence, success of the Bt brinjal partnership is likely to affect the future of other GE crops in Bangladesh, as well as other parts of the world where biotechnology is needed for food security and environmental safety.

## Introduction

### The problem

*Solanum melongena* L. (eggplant, also known as brinjal in Bangladesh) is an important, inexpensive, and popular vegetable in Bangladesh, second only to potato in production. It is grown on nearly 50,000 hectares. Its production provides an important source of cash income for small resource-poor Bangladeshi farmers. The biggest constraints to eggplant production are chronic and widespread infestations by the eggplant fruit and shoot borer (EFSB), *Leucinodes orbonalis* Guenée (Lepidoptera: Crambidae). Caterpillars damage eggplant shoots and flowers, but the most serious damage is caused by their boring into the fruit and rendering it unmarketable. Farmers routinely spray broad-spectrum insecticides, often two to three times per week, and, in some cases, twice a day. Consequently, over 100 sprays per season may be applied, resulting in high residues on the fruit. Farmers lose anywhere from 30 to 60% of the crop yield to EFSB despite the high use of insecticides. The cost of insecticide treatments accounts for 35 to 40% of the total cost of cultivation of brinjal. Such an insecticide-dependent strategy poses both environmental and health concerns.

### Creating a solution

The India-based Maharashtra Hybrid Seed Company (Mahyco) used a *Bacillus thuringiensis cry1Ac* gene to transform brinjal to be resistant to EFSB (Shelton et al., 2017). The *cry1Ac* gene is widely used in Bt cotton and the protein is a component of many organic biopesticides. In all cases, Cry1Ac has a long history of safe use (ILSI CERA, 2010). The resulting GE Bt eggplant (termed “*event*” EE-1) demonstrated control of EFSB and was provided to the Bangladesh Agricultural Research Institute (BARI) through a public private partnership between Mahyco, Cornell University, Sathguru Management Consultants, BARI and the United States Agency for International Development (USAID) under the Agricultural Biotechnology Support Project II cooperative agreement (ABSPII; http://absp2.cornell.edu). BARI subsequently introgressed the EE-1 event into nine local eggplant lines. Breeding and efficacy trials were conducted beginning in 2005 and continue today. The ABSPII project ended in 2014. A new cooperative agreement was awarded in 2015 to scale the improved Bt eggplant to Bangladesh farmers under the South Asia Eggplant Improvement Partnership (SAEIP)(http://bteggplant.cornell.edu).

## Approval and adoption of Bt brinjal

### Approval process

BARI applied to the National Technical Committee on Crop Biotechnology (NTCCB) to release Bt eggplant. Following the recommendation from NTCCB, the application for release was forwarded to the NTCCB Core Committee followed by the National Committee on BioSafety (NCB). The Bangladesh government granted approval for release of four varieties (BARI Bt brinjal varieties 1, 2, 3, and 4) for “limited cultivation” in the field on 30 October 2013 (three other varieties are pending and two others are uncertain). On 22 January 2014, Bt seedlings of the four lines were distributed to 20 farmers in four districts.

### Rapid adoption

**Figure 1:**
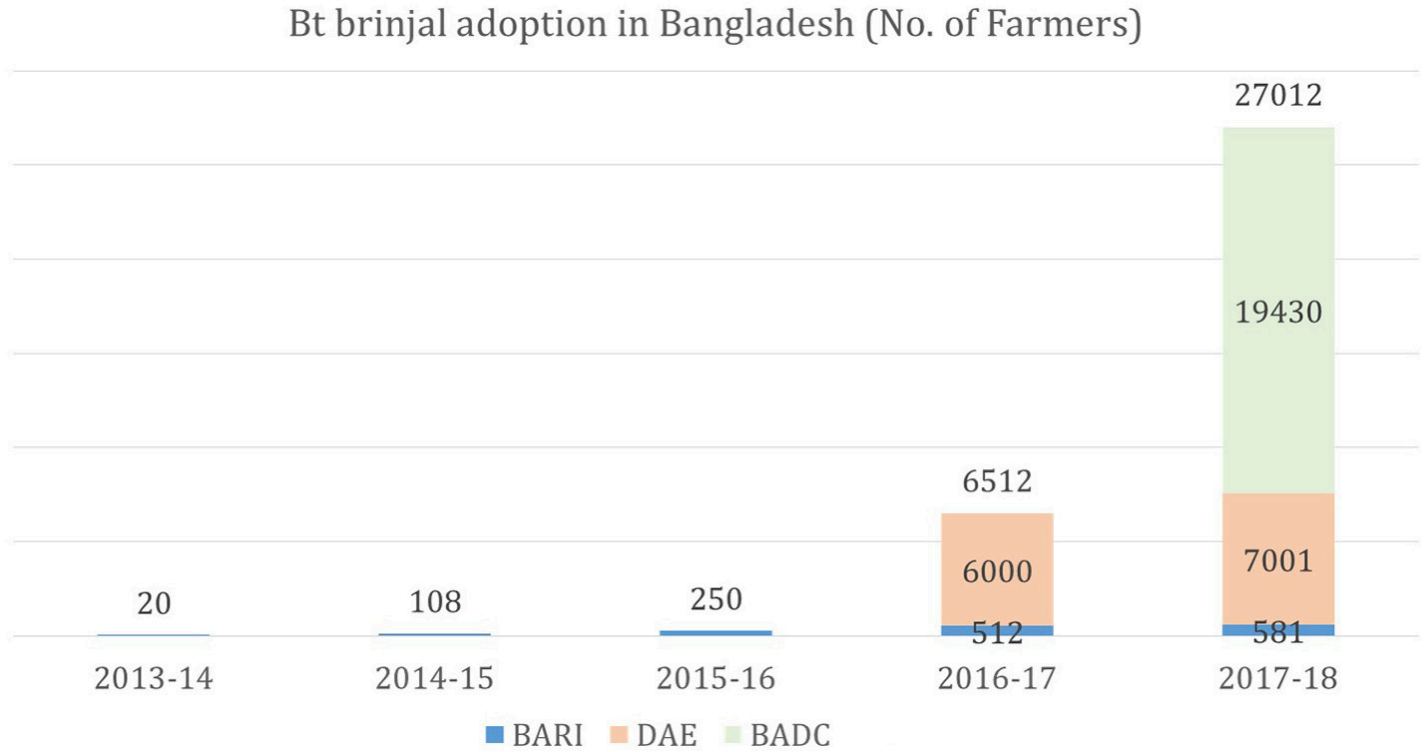
Yearly adoption rate of Bt brinjal in Bangladesh.

In 2014–15, BARI provided seeds or transplants to its On-farm Research Division (OFRD) to conduct research/demonstration trials on 108 farmer fields in 19 districts. In 2015–16 and 2016–17, demonstration trials were conducted in 250 farmer fields in 25 districts and 512 farmer fields in 36 districts, respectively. In 2017–18, BARI provided seeds to 569 farmers in 40 districts. In addition to distribution by BARI, seeds have also been distributed to farmers through the Department of Agricultural Extension (DAE) to 6,000 and 7,001 farmers in 2016–17 and 2017–18, respectively, and for sale through the Bangladesh Agricultural Development Corporation to an additional 17,950 farmers in 2018 (Figure 1). With an estimated 150,000 brinjal farmers in Bangladesh, the 2018 adoption translates to an estimated ~17% of brinjal farmers in Bangladesh who are enjoying the benefits of the technology.

## Performance

### Control of EFSB and effects on non-target arthropods

According to BARI reports for 2015 and 2016, the performance of Bt eggplant in demonstration trials was far superior to non-Bt eggplant, with fruit infestations in Bt eggplant ranging from 0.04–0.88% compared to 48–57% in non-Bt eggplant (Mondal et al., 2016).

A separate 2-year experiment (2016–17) conducted by BARI scientists compared the four Bt lines to their isolines, with and without insecticide treatments. Fruit infestation for Bt varieties varied from 0 to 2.27% in 2016, 0% in 2017, and was not significantly affected by the spray regime in either year (unpublished). In contrast, fruit infestation in non-Bt lines reached 36.70% in 2016 and 45.51% in 2017, even with weekly spraying. However, maximum fruit yield was obtained from sprayed plots compared to non-sprayed plots, indicating that other insects including whiteflies, thrips and mites can reduce plant vigor and subsequent fruit weight. This result is not unexpected since, as with other Bt crops, the EE-1 event was not designed to control all insect pests. This trial also assessed potential effects of these Bt brinjal lines on non-target arthropods. Based on other similar studies (Naranjo, 2014; Navasero et al., 2016), it is not surprising that statistically similar densities of non-target arthropods, including beneficial arthropods, were observed in both Bt and non-Bt varieties in most cases.

### Economics and pesticide use

A study was conducted by BARI scientists in 35 districts during the 2016–17 cropping season using 505 Bt brinjal farmers and 350 non-Bt brinjal farmers (unpublished). Net returns per hectare were $2,151/ha for Bt brinjal as compared to $357/ha for non-Bt brinjal, a 6-fold difference. This study also indicated that farmers saved 61% of the pesticide cost compared to non- Bt brinjal farmers, experienced no losses due to EFSB, and received higher net returns.

Similar economic benefits were obtained in a two-year experiment by another set of BARI scientists who found that a higher return was obtained from the Bt varieties over non-Bt isolines, irrespective of insecticide spray regime (unpublished). Results indicated that high quality EFSB-free brinjal could be produced without insecticide treatments but that insecticide control of “sucking insects” provided even higher economic returns on the Bt lines.

## Strategies for sustaining the technology

The economic and environmental benefits of Bt brinjal are clear: enhanced control of a difficult insect pest; reduced use of insecticides and their effects on applicators, consumers, and non-target organisms in the environment; and increased revenue to farmers. Stewardship strategies are needed to sustain these benefits in Bangladesh.

### Stewardship

Bt eggplant was the first GE crop released for cultivation in Bangladesh and, accordingly, has provided regulators and farmers with their first experience in managing a GE crop. BARI was designated as the lead Bangladesh institute to produce and distribute Bt brinjal to farmers, and partnered with the SAEIP to ensure stewardship of Bt brinjal in both the pre- and post-launch phases. To ensure long-term durability and success of the technology, the partnership has prioritized efforts to train stakeholders, including researchers, academics, seed production experts, extension professionals and farmers on the need for effective stewardship for Bt brinjal. The team at BARI has been trained on “Excellence Through Stewardship”^1^ (ETS), a life cycle approach to GE product management, primarily through the private sector partner, Mahyco, who has extensive experience in managing scaling activities commercially. This includes capacity building efforts to ensure that quality seed—genetic purity, high viability and expression of Cry1Ac—is produced in adequate amounts to meet grower demand.

Other capacity building efforts include development and documentation of standard operating procedures (SOP) for seed testing, proper seed packaging and labeling, and record keeping that meets industry audit standards. The partnership has introduced tools to monitor the production, distribution and inventory management of Bt brinjal seeds as part of the goal to meet international standards. BARI is the sole producer of seed for the four approved Bt brinjal varieties, but has recently provided breeder seed to the Bangladesh Agricultural Development Corporation (BADC) to further increase seed quantities. BARI has distributed seed for free to growers but BADC charges a minimal fee. Of note is that the four Bt brinjal lines that have been released are not hybrids, so growers can save seed, although they are discouraged from doing so for agronomic reasons. Inclusion of the EE1 event in a hybrid background would further increase the yield potential of Bt eggplant.

While stewardship begins with quality seed, other practices are equally vital for the long-term sustainability of Bt brinjal in Bangladesh. Studies have shown that plants derived from EE-1 can be considered as “high expression” plants (Hautea et al., 2016), typically a major component of an effective insect resistance management (IRM) program (Bates et al., 2005). A second common component of IRM is to utilize a refuge of non-Bt plants so that Bt-susceptible alleles can be maintained in the EFSB population. The refuge requirement was set by the partnership at 5% during this initial phase of adoption. A third component of IRM is to develop and utilize baseline studies of susceptibility to Cry1Ac and monitor for any changes that might indicate emerging resistance. Efforts are underway to enhance the existing dataset. A fourth component of IRM is pyramiding Bt genes into plants. While it is recognized that introducing pyramided plants initially would have been desirable, this was not possible when the partnership began, but is being strongly advised for the future.

### Farmer training

Farmer training is the lynchpin of sustainable production of this valuable product in Bangladesh. Prior to the first release of Bt brinjal in 2014, farmer training was conducted by BARI, and BARI continues to be the institution responsible for training. Bangladeshi farmers are well versed in growing brinjal, so training is focused on the unique aspects of Bt brinjal—mainly the requirements to plant a refuge of non-Bt brinjal and the need to manage other “sucking insects.”

BARI continues training efforts through hundreds of OFRD farm trails mentioned above in dozens of districts in Bangladesh where brinjal is grown. In addition to BARI, the Department of Agricultural Extension (DAE) and the Agriculture Information Service (AIS) have more recently become involved in training and distributing information on Bt brinjal. These units have their own facilities and personnel for farmer training. The partnership is in a position to help support their efforts to meet this increased demand for information.

### Communication efforts

Not surprisingly, the introduction of Bt brinjal has a strong following in the domestic and international media. Farmer use and satisfaction with Bt brinjal is reflected in the increasing number of positive press releases. Proactively the partnership has worked to provide access to press releases highlighting factual information for stakeholders such as the 2016 studies conducted in the Philippines that showed nearly 100% control of EFSB by Bt brinjal (Hautea et al., 2016) and no negative impacts on non-target arthropods (Navasero et al., 2016). Publishing similar results from agronomic studies and socioeconomic studies conducted in Bangladesh (as described above) is a high priority for the project. Such information will also be highlighted in the partnership website (Bteggplant.cornell.edu) which is actively maintained, and through social media. The partnership has developed print and audio-visual materials for information sharing and awareness building. It has also conducted a number of national level workshops to engage stakeholders and policy makers.

The partnership also works closely with the Cornell Alliance for Science (allianceforscience.cornell.edu) which provides factual information about agricultural biotechnology and has been a valuable partner in disseminating information about the partnership. The Alliance has enhanced capacity in social media that benefits the partnership in the short and longer term. Other activities include supporting the global March for Science to increase knowledge about agricultural biotechnology and the role it can play in ensuring food security and environmental protection.

Most importantly, the partnership continues to receive strong support from the Honorable Agriculture Minister Begum Matia Chowdhury, MP. Her words from a workshop held in March 2017 in Bangladesh have made her position clear: “Development of brinjal fruit and shoot insect resistant-Bt brinjal is a success story of local and foreign collaboration. We will be guided by the science-based information, not by the nonscientific whispering of a section of people. Good science will move on its own course keeping the anti-science people down. As human beings, it is our moral obligation that all people in our country should get food and not go to bed on an empty stomach. Biotechnology can play an important role in this effort.”

### Personnel and USAID

As with any partnership, quality personnel are essential. In the last year our partnership has benefited greatly by a new country coordinator who is well respected as a Bangladesh scientist and within the various Bangladesh agencies involved with the project.

Additionally, a new stewardship coordinator well versed with ETS became part of the team.

USAID is committed to supporting countries that wish to develop and commercialize science-based technologies including GE crops. Bangladesh chose to develop a partnership, first with ABSPII and now SAEIP, to develop and commercialize Bt brinjal to alleviate the overuse of pesticides on this important Bangladeshi crop. The partnership is well positioned to continue to increase adoption and stewardship, as well as evaluate the significant socioeconomic impacts of this technology including the positive human and environmental effects of reduced pesticide treatments. The ultimate goal is that the process and knowledge of this partnership be incorporated into the core practices of the public sector of Bangladesh and the private sector that sells and develops high quality seed.

### The regulatory system in Bangladesh

Bangladesh has a variety-based registration system rather than an event-based system. Thus, the currently approved Bt EE-1 derived lines, and the five others that were developed, must individually be approved after being tested in the field. In contrast, most other countries rely on an event-based approval process. The efficiency and cost of event-based registration helps move a product to market more rapidly. Numerous studies have shown this process does not compromise efficacy or safety. Discussions are underway in Bangladesh that may help them adopt an event-based system to allow more rapid development of lines developed by BARI and the private sector.

There is also a need to assess compliance of regulations affecting Bt brinjal at all levels from the laboratory to the field, and this could be a function of ETS. Likewise, efforts should also explore the potential use of “refuge in the bag” (i.e., a specific mix of Bt and non-Bt seed in the same container) technology to ensure farmer refuge compliance.

## Future directions for the Bt brinjal partnership

**Figure 2:**
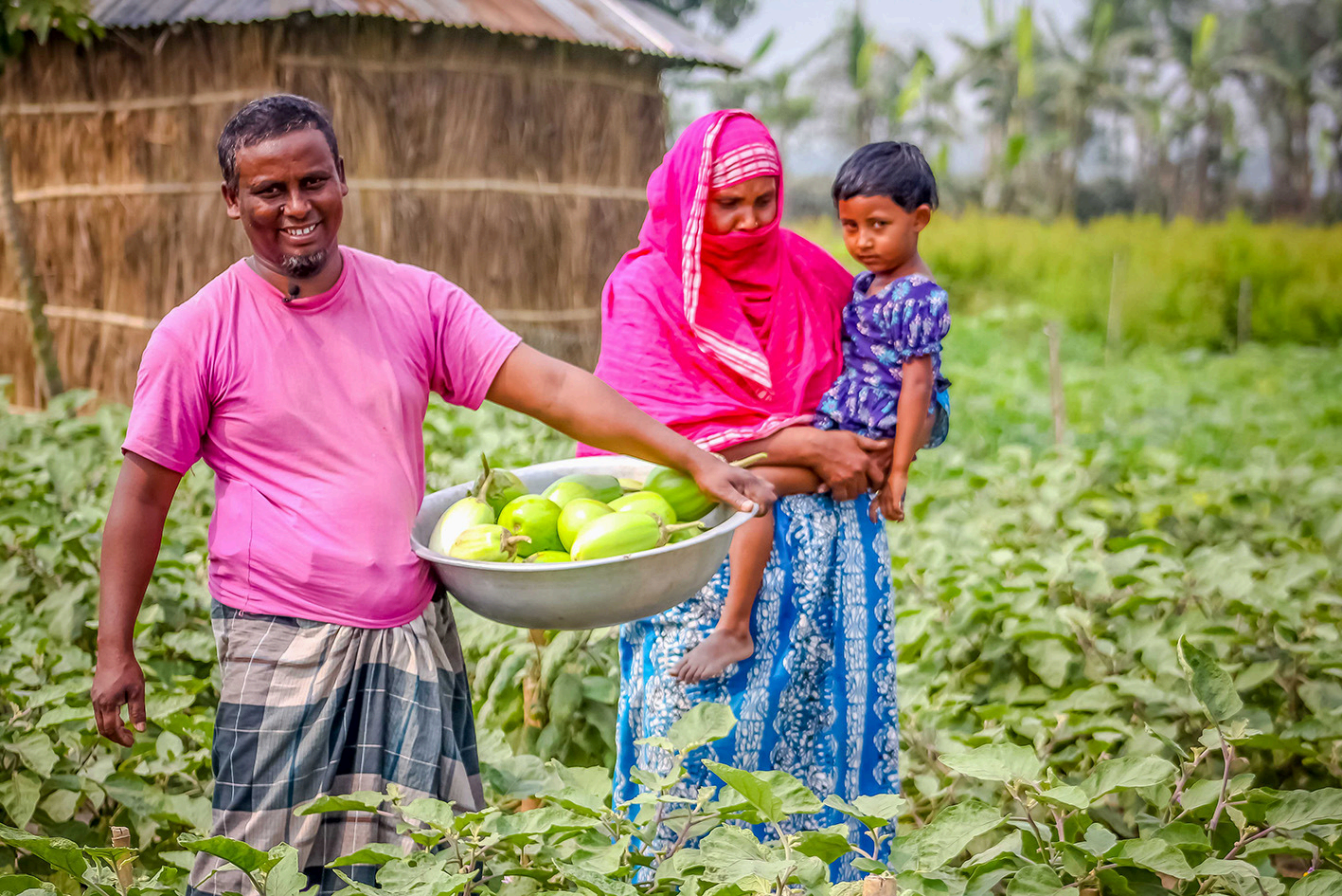
Bangladesh farmer Md. Saiful Islam and his family harvesting Bt brinjal. Photograph by Md. Arif Hossain and used with permission of photographer and the Islam family.

Although small in scale, this partnership has been vital in helping Bangladesh move Bt brinjal into farmers' fields where they will obtain the benefits (Figure 2). The partnership's role has been, and can continue to be, as a facilitator for the sustainable use of Bt brinjal primarily through capacity building and advising. While BARI is the key stakeholder in the development of the technology in Bangladesh, Bt brinjal technology has other stakeholders to carry forward the technology to the ultimate users (farmers). Other stakeholders include various government sectors (DAE, BADC, etc.), the private sector and NGOs, consumers and the media. The Ministry of Agriculture (MOA) can provide guidelines (policy and logistics) to help the partnership meet the needs of the various stakeholders. Listed below are some comments related to future directions.

- For the last several years, BARI has produced large quantities of the four Bt brinjal varieties and provided breeder seed to BADC for multiplication. As other varieties are approved, BARI can likely continue to follow this strategy until the private sector is allowed to enter the market. Meanwhile, the partnership can benefit greatly if BARI continues to focus on varietal maintenance (breeder seed production) and purification (as needed). Other activities can also enhance the partnership in the near and long term. These include: enhanced documentation of an appropriate seed-to-seed stewardship protocol; providing services such as seed testing for presence of the Bt gene to the seed multiplication agencies (currently BADC and maybe the private sector in the future); generating reliable information on Bt brinjal cultivation; communicating such to various stakeholders.
- BADC is the only public sector institution to have undertaken commercial multiplication and widespread distribution of Bt brinjal seeds in Bangladesh. The partnership believes this is a positive step to help meet the increasing demand for Bt brinjal. But, along with increased seed production, there should be appropriate information provided to farmers about the technology itself and its agronomic and stewardship requirements. BARI is in an ideal position to help meet this critical need.
- As with any new technology, stewardship is vitally important and this is true of Bt brinjal. Planting borders of non-Bt brinjal as a refuge is critical for the sustainability of the Bt brinjal technology. DAE, as the sole extension arm of the government, is in an excellent position and can play a significant role with technical support from BARI and the project to ensure planting of such refuge crops at the farmers' level. Information about using refuges should be incorporated in extension materials (booklets/leaflets) and training of field staff. Extension material and trainings should also emphasize that Bt brinjal only controls EFSB and that other pests will require supplemental control
- The private sector has considerable experience developing and bringing GE crops to market while meeting the regulatory requirements of a particular country. In most cases, (except GE papaya in Hawaii), it has been a company that has produced seeds for the GE crop and then sold them to farmers. The private sector (including NGOs involved in commercial seed operations) has not yet but could be a significant partner in the long- term development of Bt brinjal in Bangladesh and future crop innovations. In the seed industry, the private sector is considered to be efficient at developing and scaling quality seed. Once the Bt brinjal technology is made available to the private sector for commercial multiplication, the private sector may readily move forward to develop their own Bt varieties, including hybrids.
- Since BARI is a public research institution, the MOA remains the ultimate authority to determine the way forward for Bt brinjal at the farmers' level. The technology was approved four years ago, and our partnership is in a good position to work with the MOA to develop a well-defined work plan for full commercialization. A comprehensive work plan with appropriate roles and responsibilities of different stakeholders will help ensure the sustainably of Bt brinjal. The event developer (Mahyco) and other stakeholders in the private sector should be part of this discussion. The role, as well as the responsibility, of the MOA in guiding the way forward of the technology is pivotal. We believe that our project can help facilitate this plan.

## The role of Bt brinjal in Bangladesh and the world

As the first GE crop in Bangladesh, Bt brinjal plays a vital role in the future of biotechnology. The success of this first crop has set the stage for others to come. Fortunately, Bt brinjal has gotten off to a good start with increased yearly adoption and very favorable socioeconomic benefits.

The development and regulation of GE crops in Bangladesh is largely based on agricultural and scientific questions. Their advancement is made possible because the government and people of Bangladesh have embraced science-based technologies that can improve the socioeconomic well-being and environmental safety in their country. Fortunately, the Honorable Agriculture Minister Begum Matia Chowdhury, MP has been essential in making GE crops a reality in Bangladesh. Such support is needed in other parts of the world if the potential benefits of these technologies are to be realized. The success of Bt brinjal in Bangladesh should serve as an example of what can be accomplished with science-based technologies.

## Author contributions

All authors participated in the drafting of this paper as individual subject matter experts in their fields, and the authors are solely responsible for the content. Any views expressed in this paper are the views of the authors and do not necessarily represent the views of any organization, institution, or government with which they are affiliated or employed.

### Conflict of interest statement

The authors declare that the research was conducted in the absence of any commercial or financial relationships that could be construed as a potential conflict of interest.

## Footnotes

^1^http://www.excellencethroughstewardship.org/
